# The role of iatrogenic foraminal stenosis from lordotic correction in the development of C5 palsy after posterior laminectomy and fusion

**DOI:** 10.1186/s13018-015-0297-2

**Published:** 2015-10-06

**Authors:** Daniel J. Blizzard, Michael A. Gallizzi, Charles Sheets, Mitchell R. Klement, Lindsay T. Kleeman, Adam M. Caputo, Megan Eure, Christopher R. Brown

**Affiliations:** Department of Orthopaedic Surgery, Duke University Medical Center, Box 3000, Durham, NC 27710 USA; Spine Center, OrthoCarolina, Charlotte, NC USA

**Keywords:** C5 palsy, Post-operative palsy, C5, Lordosis, Cervical lordosis, Foraminal stenosis, Iatrogenic, Iatrogenic stenosis, Iatrogenic foraminal stenosis

## Abstract

**Background:**

Post-operative C5 nerve root palsy is a known complication following cervical spine surgery. Although several theories have been proposed, there remains no consensus as to the etiology of the palsies. Multiple pre-operative radiographic measures have been assessed for utility in predicting palsy. The purpose of this study is to evaluate published radiographic parameters as well as specifically evaluate the effect of cervical lordosis in the development of C5 palsy to establish thresholds that reliably predict the incidence.

**Methods:**

This study is a retrospective review of 54 consecutive multilevel cervical laminectomy and fusion surgeries performed by a single spine surgeon between June 2007 and February 2014. Pre-operative MRI and pre- and post-operative plain films were assessed to measure anteroposterior diameter (APD) of the spinal cord, cervical laminar angles, anteroposterior foraminal diameters (FD), cervical curvature index (Ishihara), cervical spine angle (C2-7), and C4-5 angle. Univariate analysis through independent *t* tests was used to compare differences between groups. Stepwise logistic regression was performed to identify pre-operative variables associated with C5 palsy. Receiver operating characteristic curves were created for significant variables to assess predictive accuracy through determining the area under the curve.

**Results:**

There were 13 (24 %) palsies in the 54 patients in the study. All palsies completely resolved within 6 months. Among pre-operative measures, FD and APD were significantly different between the palsy and non-palsy groups. The average post-operative C4-5 angle was significantly different between the groups, though the cervical spine angle and curvature index, as well as the change in these measures from pre-operative measurements, did not differ significantly between groups.

**Conclusions:**

Post-operative palsy is likely a result of iatrogenic nerve root compression from a decreased in cross-sectional area of the neuroforamen in a patient with pre-operative narrowing of the foramen. However, spinal cord drift back may also play a role from the combined effect of posterior decompression from laminectomy and relative slack afforded by increased lordosis. Accordingly, increased post-operative lordosis would increase the likelihood of effect from both of these mechanisms. We recommended limited conservative lordotic correction in patients with pre-operative foraminal narrowing.

## Background

Posterior cervical laminectomy and fusion (PCLF) is a well-established treatment for cervical spondylotic myelopathy refractory to conservative management. Numerous studies and meta-analyses have demonstrated that PCLF can reliably improve myelopathic symptoms and provide mechanical stability [1–6]. However, there are known complications with this procedure including junctional kyphosis, post-laminectomy instability, and neurological deterioration. C5 palsy remains a relatively common complication of this procedure with a published incidences ranging from 0–30 %, with a recent meta-analysis calculating an estimated rate of 6.7 % [7, 8].

The etiology underlying the development of C5 palsy remains undetermined. Proposed theories include root traction from posterior drift of the cord, iatrogenic intra-operative injury, ossification of the PLL, spinal cord ischemia, and, more recently, foraminal stenosis [9–15]. Many authors have sought to identify pre-operative symptoms and radiographic anatomical measures that can predict the likelihood of developing a palsy. Among radiographic measures, anteroposterior diameter (APD) of the cord, foraminal diameter (FD), and cord-lamina angle (CLA) have all been evaluated as potential predictive measures [8, 16–19], and a recent study by Lubelski et al. even combined these measurements in a nomogram for predicting post-operative palsy [20]. Additionally, others have proposed that technical factors of the surgery play a role in the development including width of laminectomy trough, concurrent foraminotomies, and number and location of levels included in the construct. With a wider, more extensive laminectomy, there is less posterior confinement of the cord permitting excessive posterior drift [11].

To date, no study has evaluated the role of lordosis in the development and prediction of post-operative palsy. Similar to an extensive laminectomy, increased cervical lordosis would theoretically permit further posterior drift as slack develops in the cord. Additionally, the fulcrum of flexion and extension in the spine is the posterior vertebral body [18, 21, 22]. Accordingly, increasing cervical lordosis compresses the posterior elements of adjacent vertebrae and the vertical height of the neuroforamen. The purpose of this study was to investigate cervical lordosis as a predictor of post-operative C-5 palsy given that lordosis is a measure that can be both assessed and modified intra-operatively. Our hypothesis was that both the magnitude of change from pre-operative alignment and the absolute amount of post-operative cervical lordosis would positively correlate with the development of C5 palsy.

## Materials and methods

### Subjects

After institutional review board approval, a retrospective review was conducted of patients treated by a single surgeon from July 2007 to March 2012 at a Level 1 Academic Center. Patients with a primary International Classification of Diseases (ICD) diagnosis of cervical spondylotic myelopathy who subsequently underwent multilevel cervical laminectomy with fusion were identified by Current Procedural Terminology (CPT) code from our institutional database. Further study inclusion criteria consisted of pre-operative MRI of the cervical spine, pre-operative and post-operative upright anteroposterior and lateral cervical radiographs, pre-operative and post-operative Japanese Orthopaedic Association (JOA) scores, Neck Disability Index (NDI), short form-12 mental (SF-12 M) and physical (SF-12P) composite scores, and visual analog pain scores for neck (VAS-N) and arm (VAS-A). The JOA, NDI, and SF-12 outcome questionnaires are given to all of our patients as a matter of standard practice. Exclusion criteria included any prior cervical spine surgery, presence of cervical tumor, infection, or fusions not including the C4-5 level. These criteria yielded a total of 54 patients.

The medical records for all patients meeting the inclusion criteria were accessed and reviewed. History and physical exam documentation was assessed to confirm the diagnosis of myelopathy (gait disturbance, fine motor ataxia, abnormal reflexes, motor/sensory deficits) and failure of non-operative management. Additionally, the post-operative records were reviewed for severity, chronicity, duration, and laterality of C5 palsy. Post-operative palsy was defined as a decrease in motor strength of the deltoid or biceps of at least two motor muscle grades from pre-operative examination.

### Surgical technique

Lateral mass screws, pedicle screws, and rods were used for instrumentation utilizing a standard midline posterior approach. Laminectomy was performed with a 4 mm round tip bur creating a trough at the interlaminar “V” and completed using a 1 mm Kerrison. Facet joints were then decorticated, and morselized autograft and allograft bone was subsequently packed into the facet joints along the lateral masses. The instrumentation used in all of the surgeries was manufactured by NuVasive, Inc. (San Diego, CA, USA). No concurrent foraminotomies were performed.

### Outcome evaluation

**Fig. 1:**
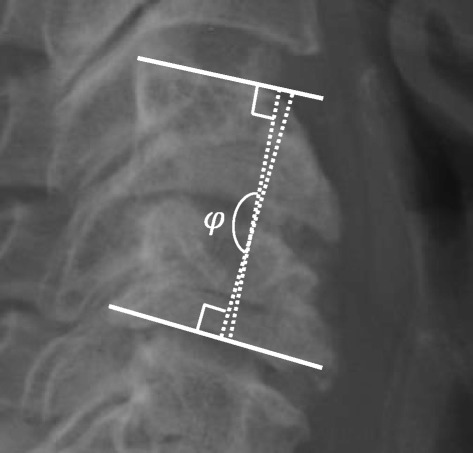
C4-5 Segmental lordosis: the angle between the superior endplate of C4 and the inferior endplate of C5

**Fig. 2:**
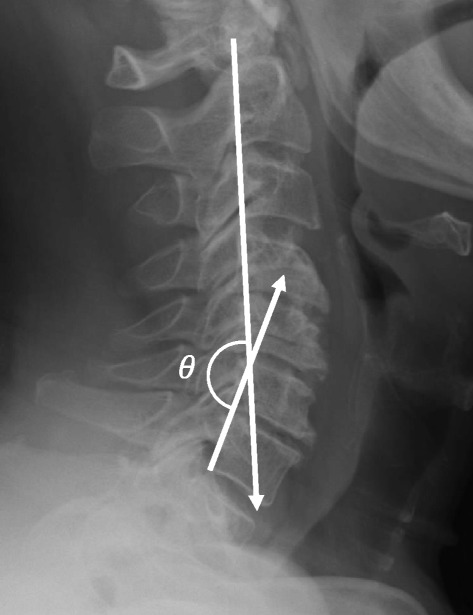
Cervical spine angle: the angle of the intersection between lines parallel to the posterior vertebral bodies of C2 and C7

**Fig. 3:**
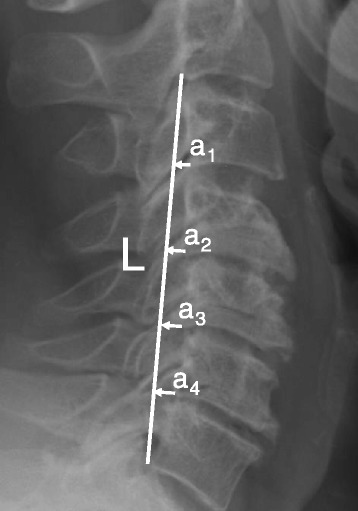
Ishihara Index: defined by the equation $\mathrm{Index}=\frac{{\displaystyle \sum a1+a2+a3+a4}}{L}\times 100$ (where a_1–4_ are the distances between the posterior margins of the inferior endplates of the vertebral bodies and a line between the posterior aspects of the inferior endplates of C2 and C7

A pre-operative MRI including axial and sagittal T1, T2, and proton-density (PD) sequences was used to measure APD of the spinal cord, bilateral FD, and CLA at the C4-5 level [20] (Fig. 1). These radiographic measures have been shown to correlate with C5 palsy in previous studies [17, 20, 23]. Additionally, pre- and post-operative (within 2 weeks of procedure) upright anteroposterior (AP) and lateral plain film radiographs were used to measure C4-5 segmental lordosis (top of C4 body to bottom of C5 body) (Fig. 1), cervical spine angle (angle between posterior aspects of C2 and C7) (Fig. 2), and cervical curvature index (Ishihara) [24] (Fig. 3). Radiographic measurements were taken independently by a senior orthopedic surgery resident (DJB) and a spine surgery fellow (MAG). Specific training for this study was consisted of simultaneous, combined review of 10 patients not included in this study using the aforementioned measures by authors DJB and MAG. The reviewers were blinded to all patient identifiers and whether each patient developed a palsy. Furthermore, the reviewers were not familiar or involved in any aspect of the care of the patients in this study, thus, eliminating any potential recall bias. Finally, all measurements were subsequently repeated by author LTK to assess inter-rater reliability. Like the other reviewers, author LTK was also blinded to all patient identifiers as well as whether a particular patient developed a palsy.

Clinical outcome scores were obtained pre- and post-operatively. Patient-reported outcomes scores included the JOA score, NDI, SF-12 M, and SF-12P composite scores and VAS-N and arm VAS-A scores.

The JOA is a four-component quantitative scale assessing myelopathy severity: (1) motor function in the arms, (2) motor function in the legs, (3) sensation, and (4) bladder function. Scores range from 0 to 17, with lower scores correlating with higher disability [25]. An improvement of 2 points has been described as clinically important [26, 27]. The NDI is a measurement cervical spine-related disability and has been demonstrated to be both reliable and internally consistent [28]. Scores range from 0 to 50, with a higher score correlating with higher disability. The SF-12 is a measure of overall health and is composed of mental (SF-12 M) and physical (SF-12P) composite scores ranging from 0 to 100, with higher scores indicating a better state of health. The VAS is pain scale ranging from 0 to 10 with higher scores representing a higher level of discomfort. Clinical outcome measures were obtained pre-operatively and at last clinical follow-up.

### Statistical analysis

Pre- and post-operative variables were assessed for their relationship to C5 palsy status. For each patient, the maximum (for pain level) or minimum (for foraminal diameter) value for each level was established and examined. Univariate analysis was performed through independent *t* tests to compare differences between groups. For pre-surgical variables, stepwise logistic regression was performed to identify variables associated with onset of C5 palsy. Receiver operating characteristic (ROC) curves were created for significant variables to assess predictive accuracy through determining the area under the curve (AUC). Inter-rater reliability was analyzed using the intra-class correlation coefficient, model (2,1) [29]. Statistical analysis was carried out using the R statistical software package version 3.0.2 (R Foundation for Statistical Computing, Vienna, Austria).

## Results

There were a total of 54 subjects (35 % female), with an average age of 58.5 (±10.5) years. Demographic variables are included in Table 1.

**Table 1:** Demographic variables

Baseline variable	No palsy		C5 palsy		*p* value
Age	57.73 ± 11.55		61 ± 8.25		0.27
Race	Black	14 (35 %)	Black	6 (46 %)	0.36
	Hispanic	1 (3 %)	Hispanic	0 (0 %)	
	American Indian	0 (0 %)	American Indian	1 (8 %)	
	Multi-race	1 (3 %)	Multi-race	0 (0 %)	
	White	24 (60 %)	White	6 (46 %)	
Gender	Male	26 (63 %)	Male	9 (69 %)	0.96
	Female	15 (37 %)	Female	4 (31 %)	
BMI	30.5 ± 7.6		30.6 ± 5.8		0.94
Smoker	Yes	12 (19 %)	Yes	0 (0 %)	0.07
	No	29 (81 %)	No	13 (100 %)	
Comorbidities^a^	3 (2,5)		4 (3, 6)		0.41
Length of stay^a^	3 (3, 5)		4 (4, 5)		0.13
Diabetes	Yes	10 (24 %)	Yes	3 (23 %)	1.0
	No	31 (76 %)	No	10 (77 %)	

^a^Reported as median and interquartile range

*BMI* body mass index

Of the 54 patients, 13 (24 %) experienced C5 palsy post-operatively (3 left, 4 right, 6 bilateral). None of the baseline demographics (age, gender, BMI, race, comorbidities, smoking, or diabetes) were predictive of developing post-operative palsy. The median length of stay was shorter in the non-palsy group (3 vs. 4 days), but there was not a statistically significant difference.

Among pre-surgical variables, right foraminal diameter (*p* = 0.04, 95 % confidence interval (CI) of difference 0.09–3.60), minimum foraminal diameter (*p* = 0.048, 95 % CI of difference 0.01–3.36), and APD (*p* = 0.006, 95 % CI of difference 0.68–3.85) were found to have a significant difference between groups, with smaller values in the palsy group. Results are summarized in Table 2. There was no difference between lordosis measures including cervical spine index, segmental lordosis, or Ishihara index for range of motion or other measured variables. Additionally, there was no significant difference in CLA.

**Table 2:** Predictive variables

Time point	Variable	No palsy	C5 Palsy	*p* value
Pre-operative	Right foraminal diameter	3.93 ± 4.99	2.09 ± 1.01	*0.04*
	Left foraminal diameter	3.91 ± 4.84	2.53 ± 0.80	*0.10*
	Minimum foraminal diameter	3.66 ± 4.87	1.97 ± 0.75	*0.05*
	Anterior posterior diameter	9.58 ± 3.76	7.32 ± 1.67	*0.006*
	Right CLA	33.43 ± 10.59	37.58 ± 8.73	0.19
	Left CLA	32.17 ± 7.43	36.61 ± 6.69	0.07
	Neutral	11.48 ± 14.77	12.37 ± 9.59	0.82
	Flexion ROM	14.77 ± 12.16	13.59 ± 11.61	0.78
	Extension ROM	−22.68 ± 15.15	−25.57 ± 11.66	0.53
	Ishihara Index	8.12 ± 25.03	2.54 ± 13.71	0.42
	Cervical Spine Angle	8.07 ± 15.41	12.08 ± 12.77	0.36
	Segmental Lordosis	0.17 ± 5.01	1.64 ± 8.06	0.54
Post-operative	Ishihara Index	−2.05 ± 6.45	3.63 ± 10.83	0.15
	Cervical Spine Angle	7.82 ± 13.12	12.07 ± 13.91	0.34
	Segmental Lordosis	−0.09 ± 5.22	2.45 ± 2.77	*0.03*

*CLA* cord-lamina angle, *ROM* range of motion. Italics signify statistically significant values P < 0.05

Among post-surgical values, segmental lordosis was significantly different between the groups, with higher values in the C5 palsy group (*p* = 0.03, 95 % CI of difference 0.27–4.80). The other measures of lordosis did not differ significantly between the groups.

Logistic regression resulted in significant values for minimum foraminal diameter (MFD) and APD, with a Nagelkerge *R*^2^ of 0.43. The diagnostic accuracy of MFD and APD was summarized in Table 3. The AUC for minimum foraminal diameter was 0.80, with a cut point of 2.4 mm (sensitivity 0.83, specificity 0.78, positive likelihood ratio 3.9). Given the baseline prevalence of 24 % in this sample, a patient with a minimum foraminal diameter of less than 2.4 mm would have a 55 % chance of developing C5 palsy. The AUC for APD was 0.81, with a cut-point of 7.6 mm (sensitivity 0.66, specificity 0.89, positive likelihood ratio 6.1). A patient with an APD less than 7.6 mm would have a 66 % chance of developing C5 palsy.

**Table 3:** Single cut-off scores and resultant post-test probabilities

	Cut-off	AUC	+LR	Post-test probability of C5 palsy
New minimum diameter	2.4	0.80 (0.64–0.95)	3.9	0.55
Anterior posterior diameter	7.6	0.79 (0.63–0.95)	6.1	0.66

*AUC* area under the curve, *+LR* positive likelihood ratio

A combination of the findings was calculated, as indicated in Table 4. If both APD and MFD were observed to be below the cut-off, the likelihood of post-operative C5 palsy in this sample was 86 %.

**Table 4:** Combination of cut-off scores

Number of positive results (below cut-off)	Sensitivity	Specificity	+LR	Post-test likelihood of palsy
1 or more	0.92	0.68	2.8	0.48
2	0.50	0.97	18.5	0.86

*+LR* positive likelihood ratio

Inter-rater reliability was analyzed through intra-class correlation coefficient demonstrating values for individual measurements ranging from 0.70 to 0.85, meeting or exceeding minimum standards for group/research-level reliability [29].

In comparing pre- to post-test measurements, the changes in index, angle, and lordosis were not significantly different between groups (Table 5). There were no statistically significant differences for self-report measures. Improvement in Neck Disability Index and maximal arm pain scores demonstrated clinically meaningful differences between groups (6.5 and 2.7, respectively), with worse scores in those developing C5 palsy, but lack of follow-up outcomes (60 % follow-up rate) resulted in insufficient power to detect a difference.

**Table 5:** Pre- to post-operative change in measures and scores

Variable	No palsy	C5 Palsy	*p* value
Neck Disability Index	4.05 ± 10.03	−2.45 ± 8.8	0.07
Maximum VAS arm	1 ± 2.75	−1.71 ± 2.93	0.06
Ishihara Index	−8.74 ± 25.2	1.09 ± 13.4	0.17
Cervical Spine Angle	0.24 ± 10.11	0.02 ± 13.93	0.96
Segmental lordosis	−1.73 ± 4.13	0.44 ± 5.74	0.30
JOA	−2.38 ± 1.41	−2.33 ± 0.5	0.92
SF-12 M	−4.24 ± 12.82	−2.16 ± 4.03	0.50
SF-12P	−5.8 ± 8.92	−3.31 ± 11.05	0.53
VAS neck	0.75 ± 3.89	0.88 ± 2.23	0.92

*JOA* Japanese Orthopaedic Association score, *SF-12 M* short form-12 Mental score, *SF-12P* short form-12 Physical score, *VAS neck* visual analog pain score for neck

All C5 palsies in this study were transient and resolved entirely by 6 months post-operatively. Post-operative C5 strength and sensation was equivalent or improved from pre-operative clinical exam testing in all patients.

## Discussion

Numerous studies have demonstrated that refractory myelopathy is reliably treated with posterior cervical laminectomy and fusion [1–6]. Among the potential complications following PCLF, C5 palsy remains the most common with severity ranging from temporary, incomplete motor, or sensory dysfunction to permanent, complete palsy. A recent meta-analysis reported a 6.7 % rate of C5 palsy after all multilevel cervical decompression procedures, while others have reported rates up to 30 % [7, 8]. There is no statistically significant difference in the rate of palsy based on surgical procedure [7]. In the current study we found a rate of 24 %.

Multiple theories for the etiology of C5 palsy development have been proposed [9–11]. The initial theory for palsy development was intra-operative, iatrogenic injury from instruments; however, subsequent studies with intra-operative monitoring did not reliably predict or prevent post-operative nerve palsy [8, 30, 31]. Others have proposed that the anterior horn of the spinal cord undergoes damage from reperfusion injury after cord decompression, but this has not been substantiated on MR imaging. Additionally, technical factors including width of laminectomy, concurrent foraminotomies, and number of included levels have been proposed as potential causes. Finally, others attribute post-operative palsy to tethering and traction of the nerve roots as the cord drifts backward once the buttress of the posterior elements is removed [32]. However, this theory does not explain the occurrence of palsy after anterior decompression.

In the study herein, we proposed that increased cervical lordosis plays an important if not pivotal role in the development of post-operative C5 palsy. The center of flexion and extension in the spine lies approximately at the posterior one-third of the vertebral body [22]. Accordingly, when the angle between adjacent vertebrae increases, there is resultant distraction between the anterior borders of adjacent vertebral bodies and compression of the posterior elements. The superior and inferior borders of the neuroforamen are defined by the inferior aspect of the superior pedicle and the superior aspect of the inferior pedicle, respectively. With increased lordosis, there is a proportional resultant reduction in the superior-to-inferior diameter and a decrease is the cross-sectional area of the foramen leading to potential iatrogenic foraminal stenosis. In the present study, patients with post-operative C5 palsy had a statistically significant greater amount of segmental lordosis (*p* = 0.007). Although the post-operative Ishihara Index as well as the change in Ishihara Index and segmental lordosis trended towards significance, these measures did not meet our definition of statistical significance (*p* < 0.05). Additionally, pre-operative anterior-posterior diameter of the neuroforamen was found to be significantly smaller in patients with C5 palsy. This is further consistent with the theory that iatrogenic compression of the neuroforamen creates foraminal stenosis as patients with a smaller initial foramen would be at increased risk of developing symptomatic foraminal stenosis after vertical compression of the foramen from increased lordosis. For this reason, multiple studies have investigated the utility of prophylactic foraminotomies to prevent post-operative C5 palsy in multilevel laminoplasties or laminectomies. Several smaller studies have demonstrated a significant difference in the incidence of palsy with prophylactic foraminotomies [33, 34], while a more recent meta-analysis of 1001 cases revealed that foraminotomies at the time of surgery can actually be a risk factor for the development of palsy [35].

Finally, we found that the anterior-posterior diameter of the spinal canal at the C4-5 level was significantly smaller in patients with C5 palsy. This is consistent with previous studies and is consistent with the proposed tethering or traction theory of palsy [32, 36]. Additionally, increased lordosis may allow further posterior translation of the cord as the relative decrease in height of the cervical spine would reduce tension in the spinal cord which is fixed in position at the cranial and caudal ends.

This study has several important limitations. No post-operative cross-sectional imaging was routinely obtained. This limits evaluation of actual posterior spinal cord drift and assessment of neuroforamen size and morphology. Additionally, the rate of C5 palsy in this study (24 %) exceeds the rate of palsy in many of the meta-analyses on the subject, but all of the palsies were temporary. As this is a single surgeon series, it is possible that operative techniques including patient positioning, lordotic correction, or width of laminectomy potentially contributed to a relatively higher rate. Furthermore, the recovery of nerve function in all patients, as opposed to reports of permanent palsy in the literature, could be attributed to varying etiologies of palsies between surgical techniques. Recovery of function in the patients in this study may result from accommodation of the nerve roots to the new foraminal morphology or loss of lordosis over time. This could not be delineated from the data available in this retrospective study.

## Conclusions

Regardless of severity or duration, post-operative C5 palsy has significant implications for functioning, rehabilitation, and patient satisfaction. Our conclusion from the results of this study is that post-operative palsy is likely a combined result of iatrogenic nerve root compression from a decreased in the vertical diameter of the neuroforamen and traction on the compresses nerve root as the spinal cord is allowed to drift back due to the combine effect of posterior decompression from laminectomy and relative slack afforded by increased lordosis. Accordingly, increased post-operative lordosis would increase the likelihood of effect from both of these mechanisms. It is our recommendation that APD of the cord and foraminal diameter should be evaluated pre-operatively, and patients with measures below the thresholds specified herein should undergo limited lordotic correction with posterior laminectomy and fusion.

## Abbreviations

APD: anteroposterior diameter of the cord
AUC: area under the curve
CLA: cord-lamina angle
CPT: Current Procedural Terminology
FD: foraminal diameter
ICD: International Classification of Diseases
JOA: Japanese Orthopaedic Association
NDI: Neck Disability Index
PCLF: posterior cervical laminectomy and fusion
ROC: receiver operating characteristic curve
SF-12: short form-12 outcome score
VAS: visual analog pain scores
